# 14-3-3 regulation of Ncd reveals a new mechanism for targeting proteins to the spindle in oocytes

**DOI:** 10.1083/jcb.201704120

**Published:** 2017-10-02

**Authors:** Robin Beaven, Ricardo Nunes Bastos, Christos Spanos, Pierre Romé, C. Fiona Cullen, Juri Rappsilber, Régis Giet, Gohta Goshima, Hiroyuki Ohkura

**Affiliations:** 1 Wellcome Centre for Cell Biology, School of Biological Sciences, University of Edinburgh, Edinburgh, Scotland, UK; 2 Institut de Génétique et Développement de Rennes, Centre National de la Recherche Scientifique, UMR 6290, Université de Rennes, Rennes, France; 3 Chair of Bioanalytics, Institute of Biotechnology, Technische Universität Berlin, Berlin, Germany; 4 Division of Biological Science, Graduate School of Science, Nagoya University, Nagoya, Japan

## Abstract

The meiotic spindle is formed without centrosomes in a large volume of oocytes. Local activation of crucial spindle proteins around chromosomes is important for formation and maintenance of a bipolar spindle in oocytes. We found that phosphodocking 14-3-3 proteins stabilize spindle bipolarity in *Drosophila melanogaster* oocytes. A critical 14-3-3 target is the minus end–directed motor Ncd (human HSET; kinesin-14), which has well-documented roles in stabilizing a bipolar spindle in oocytes. Phospho docking by 14-3-3 inhibits the microtubule binding activity of the nonmotor Ncd tail. Further phosphorylation by Aurora B kinase can release Ncd from this inhibitory effect of 14-3-3. As Aurora B localizes to chromosomes and spindles, 14-3-3 facilitates specific association of Ncd with spindle microtubules by preventing Ncd from binding to nonspindle microtubules in oocytes. Therefore, 14-3-3 translates a spatial cue provided by Aurora B to target Ncd selectively to the spindle within the large volume of oocytes.

## Abstract (precis)

14-3-3 interacts with the kinesin-14 Ncd and prevents it from binding microtubules. Aurora B provides a spatial cue that releases Ncd from 14-3-3 around chromosomes, allowing Ncd to selectively bind the spindle microtubules in the large volume of oocytes.

## Introduction

Meiotic spindle formation in oocytes faces two challenges: lack of centrosomes and a large cytoplasmic volume in oocytes (Ohkura, 2015). Spatial regulation of motor and nonmotor microtubule-associated proteins is crucial to overcoming these challenges. Furthermore, multiple kinases are important for spindle assembly and organization in oocytes (Sampath et al., 2004; Pearson et al., 2005; Colombié et al., 2008; Swain et al., 2008; Tseng et al., 2010; Loh et al., 2012; Radford et al., 2012b; Sumiyoshi et al., 2015), but little is understood of how they regulate a meiotic spindle. It has been proposed that, in addition to Ran, chromatin-bound Aurora B provides a critical spatial cue for bipolar spindle assembly in oocytes (Sampath et al., 2004; Colombié et al., 2008; Radford et al., 2012b), but little is yet known about how this signal is translated into spindle morphogenesis except that it inhibits the microtubule depolymerase kinesin-13 (Ohi et al., 2004).

14-3-3 phosphodocking proteins sit at the core of many phosphoregulatory pathways and can act as integrators of different pathways (Morrison, 2009; Gardino and Yaffe, 2011). 14-3-3 proteins bind phosphoproteins and change their activity, localization, or protein interaction. *Drosophila melanogaster* has only two 14-3-3 isoforms, ε and ζ, compared with the seven vertebrate isoforms that frequently act redundantly (Darling et al., 2005). *Drosophila* therefore provides an advantage in defining in vivo roles of 14-3-3. It has revealed roles in development (Chang and Rubin, 1997; Kockel et al., 1997; Benton et al., 2002), but a role in the meiotic spindles has not been established previously.

In this study, we report that 14-3-3 is important for maintaining a bipolar spindle in oocytes. 14-3-3 interacts with the kinesin-14 Ncd and prevents it from binding to microtubules, but further phosphorylation by Aurora B releases Ncd from 14-3-3 to allow Ncd binding to microtubules. In response to the spatial cue provided by Aurora B, 14-3-3 promotes Ncd binding specifically to spindle microtubules by preventing it from binding other microtubules in the large cytoplasmic volume of oocytes.

## Results and discussion

### 14-3-3 regulates the meiotic spindle in oocytes

**Figure 1.**
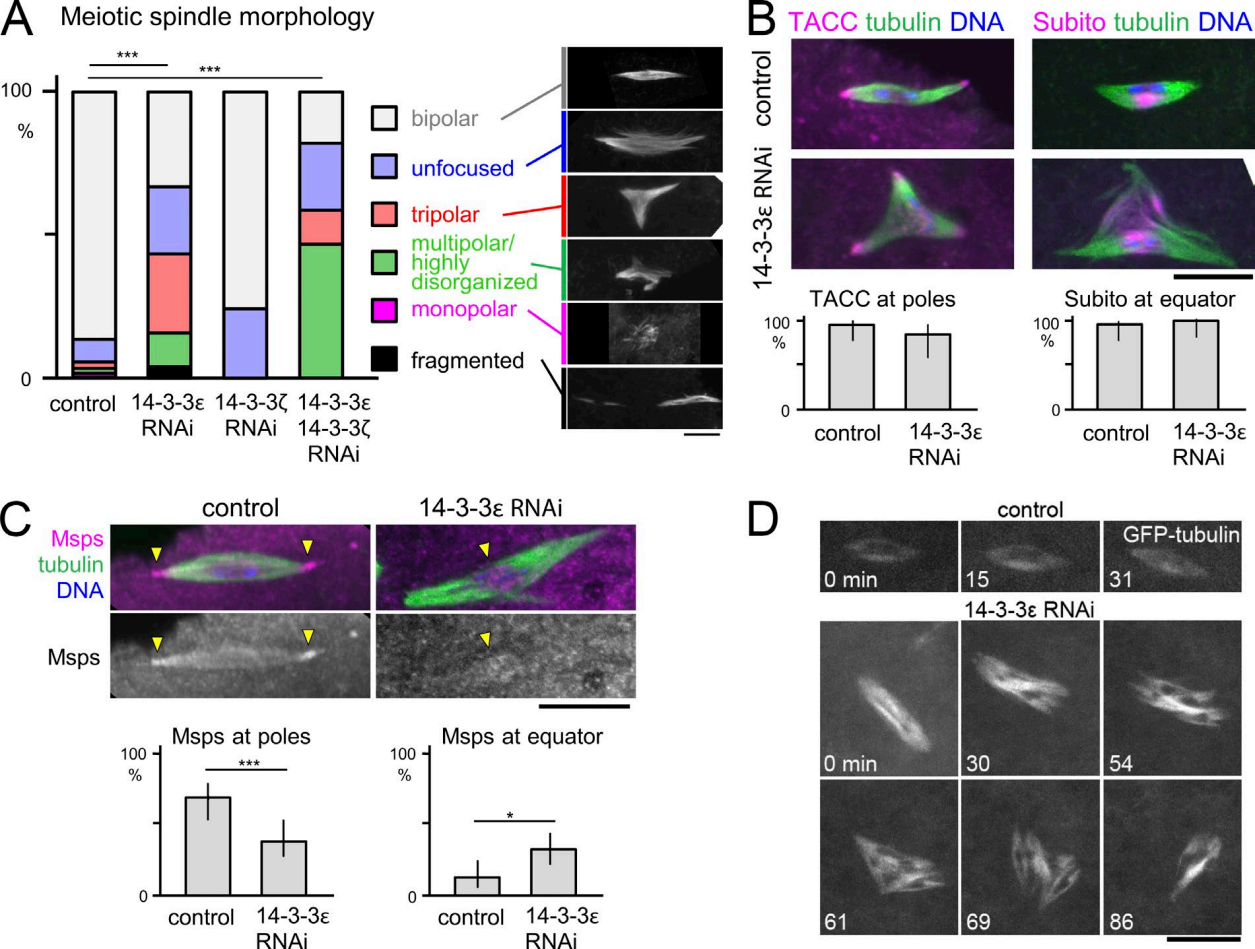
**Loss of 14-3-3 results in disrupted organization of female meiotic spindles and Msps pole localization.** (A–C) Meiotic spindle morphologies in immunostained mature metaphase-I–arrested oocytes depleted of 14-3-3. *, P = 0.013; ***, P < 0.001. *n* = 170, 174, 16, and 17 (A); *n* = 17, 17, 21, and 18 (B); *n* = 49 and 65 (C). Error bars represent 95% confidence intervals. Arrowheads in C indicate concentrations of Msps. (D) Live imaging of mature oocytes expressing GFP–α-tubulin. Bars, 10 µm.

To test a potential function of 14-3-3ε in meiotic spindle organization, we immunostained mature oocytes that naturally arrest in metaphase I. Depletion of 14-3-3ε by expressing either of two nonoverlapping shRNAs in the female germline resulted in similar abnormal spindle morphologies (Fig. 1, A–C; and Fig. S1). Interestingly, in S2 cells, 14-3-3ε has no significant roles in the morphology of mitotic spindles both in the presence or absence of centrosomes (Goshima et al., 2007; Moutinho-Pereira et al., 2013).

In contrast with 14-3-3ε, depletion of the other isoform, 14-3-3ζ, in oocytes showed no spindle organization defects (Fig. 1 A). Furthermore, 14-3-3ε depletion led to female sterility, whereas 14-3-3ζ depletion did not. Codepletion of 14-3-3ε and ζ led to a more severe spindle defect than loss of 14-3-3ε alone (Fig. 1 A), demonstrating that the two 14-3-3 isoforms together regulate the meiotic spindle with 14-3-3ε playing the major role.

### 14-3-3 maintains the bipolarity of the acentrosomal spindle in oocytes

To define the role of 14-3-3 in the meiotic spindle, matured control or 14-3-3ε RNAi oocytes were immunostained. In control oocytes, we typically observed a bipolar metaphase I spindle with focused poles (Fig. 1, A–C). In contrast, 14-3-3ε–depleted oocytes had abnormal spindles that were most typically tripolar or with unfocused poles (Fig. 1, A–C). The localization of the pole protein TACC and the equator protein Subito/MKlp2 were not altered (Fig. 1 B), suggesting that the spindle bipolarity is specifically altered. In contrast, the crucial microtubule regulator Msps (the XMAP215/TOG orthologue) that normally accumulates at the spindle poles was more often observed at the spindle equator upon 14-3-3ε depletion (Fig. 1 C). Interestingly, we previously found that a hypomorphic *msps* mutant showed a tripolar meiotic spindle morphology (Cullen and Ohkura, 2001) similar to that seen upon 14-3-3ε depletion. Compromised Msps localization is therefore likely to contribute to the spindle defect seen in 14-3-3ε–depleted oocytes.

Next, to determine whether 14-3-3ε is required for the stability of spindle bipolarity, we imaged live metaphase I oocytes expressing GFP–α-tubulin. In control oocytes, all bipolar spindles stably retained their organization (Fig. 1 D; Colombié et al., 2008). In contrast, 35% of 14-3-3ε RNAi spindles changed their morphology during the imaging for a mean of 39 min each. In some cases, a bipolar spindle became tripolar, or a tripolar spindle became bipolar. In other cases, more complex morphological changes were involved (Fig. 1 D). This revealed the important role of 14-3-3ε for the stability of bipolar spindles in oocytes.

### 14-3-3 interacts with the kinesin-14 Ncd

To identify potential binding partners of 14-3-3, we used recombinant GST–14-3-3ε for pulldown from ovary- or S2-cultured cell extract. Mass spectrometry of these fractions and controls identified potential binding partners including previously known 14-3-3 partners such as Par-1 and Bazooka (Table S1; Benton et al., 2002; Benton and St Johnston, 2003). 14-3-3ζ was also identified, suggesting heterodimer formation.

**Figure 2.**
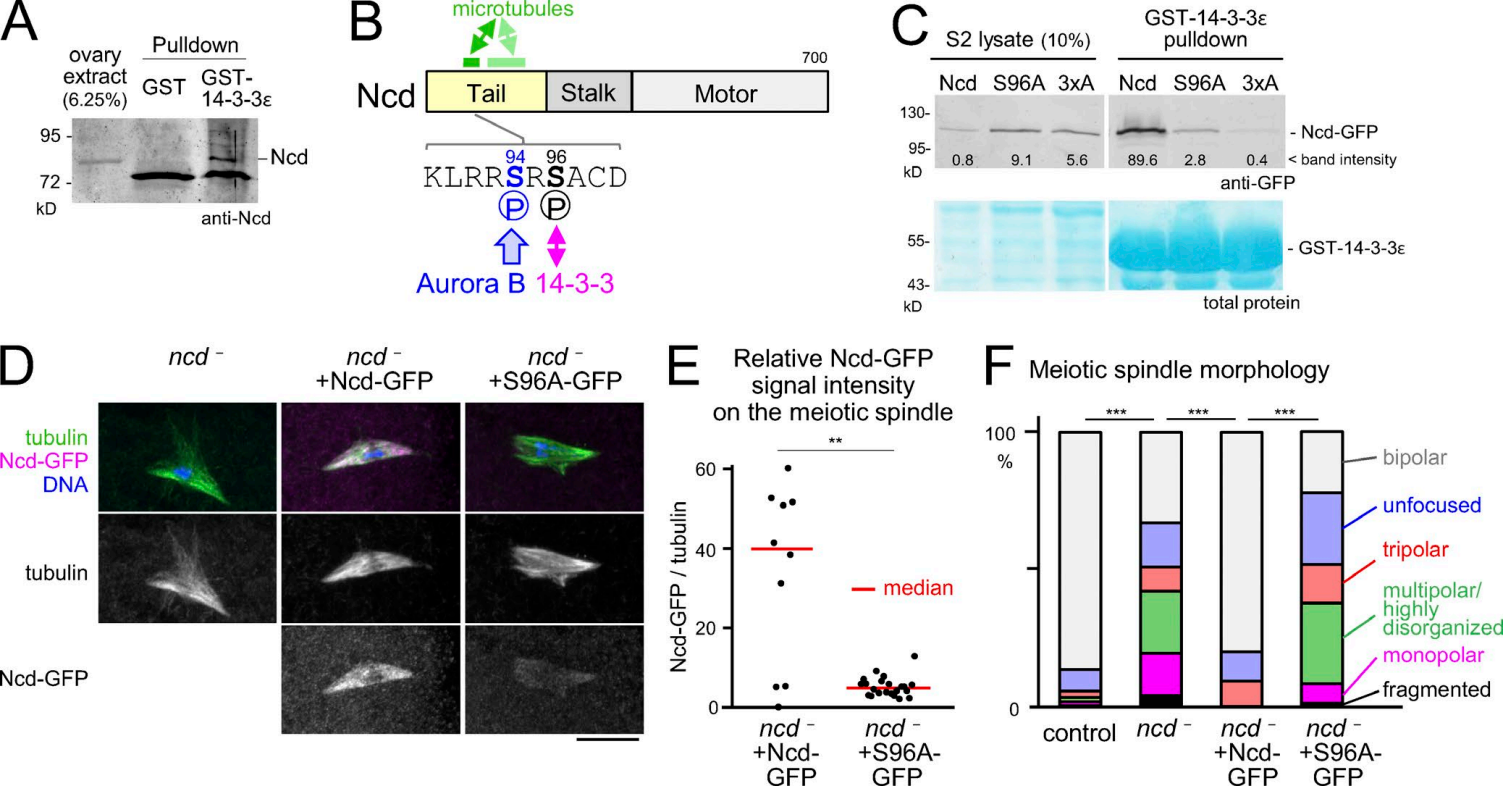
**14-3-3 binding to Ncd is important for Ncd association with meiotic spindles and spindle bipolarity in oocytes.** (A) Western blot of ovary extract and pulldown fractions with GST–14-3-3ε or GST control probed by an anti-Ncd antibody. (B) Ncd with the main 14-3-3 binding site (S96) and strong (dark green) and weaker (pale green) microtubule binding regions indicated. (C) Western blot probed by a GFP antibody and protein staining of pulldown fractions with GST–14-3-3ε from lysates of S2 cells expressing WT, S96A, or 3×A versions of Ncd-GFP, but depleted of the endogenous Ncd. (D) Immunostaining of spindles in *ncd^1^* mutant oocytes carrying no transgenes, one Ncd-GFP transgene, or two Ncd(S96A)-GFP transgenes. GFP signals were captured and modified using identical settings. Bar, 10 µm. (E) GFP signal intensity relative to α-tubulin signal on the spindle. *n* = 10 and 22. **, P < 0.01. This experiment was repeated using a different microscopy in Fig. 5 B. (F) Spindle morphologies in oocytes. *n* = 170, 45, 10, and 50. ***, P < 0.001.

In addition, previously unreported 14-3-3 partners were identified, including the kinesin-14 Ncd. Our immunoblotting confirmed that endogenous Ncd protein was pulled down by the GST–14-3-3ε from ovaries but not by the GST control (Fig. 2 A). Ncd is important for stabilizing the bipolar spindle in oocytes (Kimble and Church, 1983; Hatsumi and Endow, 1992; Matthies et al., 1996) and also for efficient localization of Msps to the poles (Cullen and Ohkura, 2001). As described in the first section of the Results and discussion, 14-3-3ε depletion resulted in compromised Msps pole localization and similar spindle defects to a hypomorphic *msps* mutant. Therefore, we hypothesized that 14-3-3 interaction with Ncd is important for stabilizing spindle bipolarity in oocytes.

To specifically disrupt the 14-3-3–Ncd interaction, we determined the interaction sites on Ncd. Bioinformatics analysis identified S96 as the site that best fits the consensus (RxxpS/pTxP; Johnson et al., 2010) and S79 and S114 as sites that fit with low stringency. All three sites are located in the nonmotor tail region of Ncd (Fig. 2 B). Previous global phosphoproteomics analyses (Bodenmiller et al., 2007; Zhai et al., 2008; Hilger et al., 2009) showed that all three sites are indeed phosphorylated in *Drosophila*.

To test the requirements of these sites for 14-3-3 binding, we made nonphosphorylatable alanine mutations of the best predicted site alone, Ncd(S96A), or of all three sites, Ncd-3×A. We first tested whether these mutations disrupt interaction with 14-3-3ε using S2 cells. We depleted the endogenous Ncd by RNAi and transiently expressed GFP-tagged WT or mutant Ncd resistant to RNAi. The amount of Ncd pulled down with GST–14-3-3ε was drastically reduced for Ncd(S96A) compared with WT Ncd, with Ncd-3×A giving minimal binding (Fig. 2 C). We conclude that phospho-S96 is a critical binding site for 14-3-3ε, although phospho-S79 and phospho-S114 may facilitate some residual binding.

### 14-3-3 binding promotes spindle association and function of Ncd in oocytes

To test for a role of 14-3-3–Ncd interaction, we expressed Ncd(S96A) in an *ncd* null mutant (*ncd^1^*) background. It has previously been shown that expression of Ncd-GFP from the native *ncd* promoter can rescue the meiotic spindle phenotype observed in the *ncd* null mutant (Endow and Komma, 1997). We therefore generated transgenic flies expressing Ncd-GFP with or without the S96A mutation under the native *ncd* promoter. We noticed a reduction in the protein levels for Ncd(S96A) compared with that of the WT form. To compensate for this, we compared one copy of the WT transgene with two copies of the mutant transgene, which gave comparable protein levels (Fig. S1 B).

The spindle signal of Ncd(S96A)-GFP relative to the α-tubulin signal was dramatically reduced compared with Ncd-GFP (Fig. 2, D and E) even when comparable amounts were expressed (Fig. S1 B). Consistent with these observations, 14-3-3–depleted oocytes also have a reduced Ncd protein level and localization on the meiotic spindle (Fig. S1, C–F). Collectively, we conclude that 14-3-3 binding to phospho-S96 of Ncd is essential for efficient association of Ncd with the meiotic spindle in oocytes.

In the *ncd* mutant without any transgenes, we observed highly frequent spindle abnormalities including spindles with multiple poles or unfocused poles (Fig. 2, D and F) as previously described (Kimble and Church, 1983; Hatsumi and Endow, 1992; Matthies et al., 1996). The WT Ncd-GFP transgene restored spindle bipolarity, whereas the Ncd(S96A)-GFP transgene did not rescue these spindle abnormalities (Fig. 2, D and F). These observations demonstrated that 14-3-3 binding to phospho-S96 of Ncd is essential for efficient Ncd association with the meiotic spindle as well as its function in organizing the meiotic spindle.

### 14-3-3 interaction suppresses binding of Ncd to microtubules in vitro

S96 of Ncd lies within a microtubule-binding region of the nonmotor tail of Ncd (Fig. 2 B; Karabay and Walker, 1999). This microtubule-binding activity of the Ncd tail is thought to be important for cross-linking parallel microtubules to focus spindle poles in oocytes (Sköld et al., 2005). Therefore, we hypothesized that 14-3-3 binding may enhance the microtubule-binding activity of the Ncd tail.

**Figure 3.**
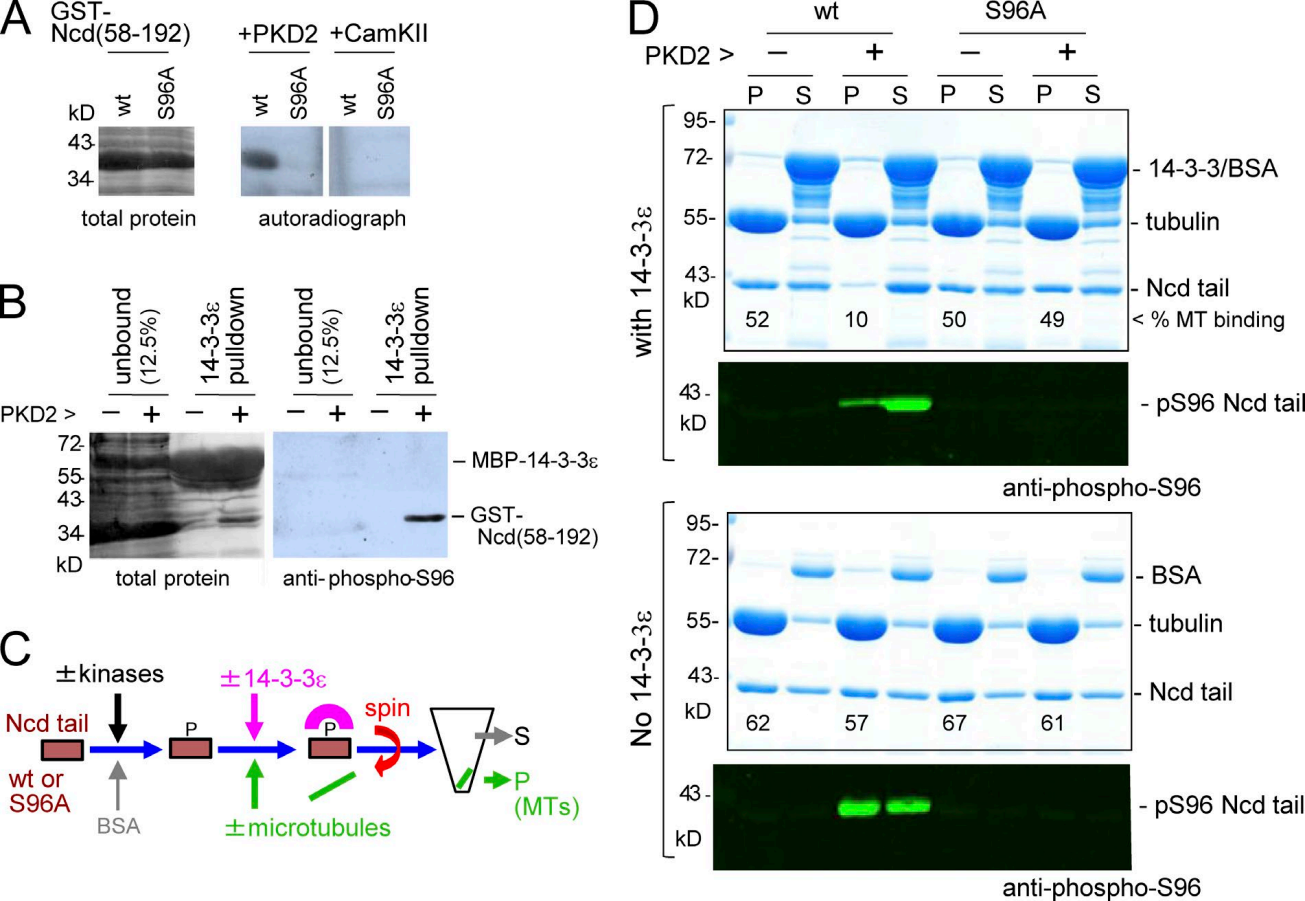
**14-3-3 inhibits Ncd microtubule binding.** (A) Kinase assay using PKD2 or CAMKIIα, Ncd(58–192) with or without the S96A mutation, and [^32^P]ATP. (B) Western blot probed by a phospho-S96 Ncd antibody and total protein staining of fractions pulled down by MBP–14-3-3ε after a kinase assay with cold ATP. (C) Microtubule (MT) binding assay. Ncd or Ncd(S96A) was incubated with PKD2 and/or Aurora B (AurB) or without and then mixed with microtubules and GST–14-3-3ε before spinning down. (D) Microtubule binding assay of the Ncd tails with or without MBP–14-3-3ε. P, pellet; S, supernatant.

To test this hypothesis biochemically, we first confirmed that 14-3-3ε can directly bind phospho-S96 of Ncd in vitro. We expressed and purified a GST-fused WT Ncd tail fragment (amino acids 58–192) and nonphosphorylatable Ncd(S96A) in bacteria. To phosphorylate Ncd at S96 in vitro, we tested commercially available protein kinases, human PKD2 and CAMK2, predicted to phosphorylate S96. We found that PKD2 but not CAMK2 can phosphorylate the Ncd tail fragment specifically at S96 in vitro (Fig. 3 A). We also generated a phosphospecific antibody against phospho-S96 and showed that 14-3-3ε specifically and efficiently pulled down the phosphorylated Ncd tail, confirming that 14-3-3ε binds to phospho-S96 (Fig. 3 B).

Next, we examined whether 14-3-3ε binding increases the microtubule binding activity of Ncd in vitro (Fig. 3 C). The purified Ncd tail was fully phosphorylated by PKD2 and then incubated with microtubules prepolymerized from porcine brain tubulin in the presence and absence of 14-3-3ε. After centrifugation, the microtubule fraction (pellet) and the supernatant were analyzed by Coomassie staining and immunoblotting using the phosphospecific Ncd(pS96) antibody.

To our surprise, we found that 14-3-3ε drastically reduced the microtubule-binding activity of Ncd phosphorylated at S96 by PKD2 (Figs. 3 D and S2). Controls (either S96 phosphorylation alone or the presence of 14-3-3ε alone) did not alter the microtubule-binding activity of this Ncd tail fragment (Figs. 3 D and S2). We therefore conclude that phosphodependent binding of 14-3-3ε to Ncd suppresses microtubule binding rather than enhancing it, which is in apparent contradiction to our cytological observations in Figs. 2 D and S1 C. To confirm that phosphorylation of S96 and not other sites is responsible for this reduction, the same experiment was performed using the fragment carrying the S96A mutation. The S96A mutation on its own or together with PKD2 incubation did not alter the microtubule binding or solubility of this mutant fragment in the presence or absence of 14-3-3ε (Figs. 3 D and S2). This confirms that phosphorylation at S96 is required for the suppression of the microtubule-binding activity by 14-3-3.

### Aurora B releases Ncd from 14-3-3ε to restore the microtubule-binding activity of Ncd

14-3-3 binding suppresses microtubule-binding activity of Ncd in vitro, whereas our cytological study showed that 14-3-3 binding promotes Ncd association with the meiotic spindle in oocytes. A key to reconcile these apparently contradictory findings could lie in the observation that the large cytoplasmic volume of oocytes contains numerous nonspindle microtubules (Radford et al., 2012a), which potentially compete with spindle microtubules for the binding of microtubule-associated proteins. We hypothesize that 14-3-3 binding suppresses the interaction of Ncd with nonspindle microtubules, but this 14-3-3 binding is prevented around chromosomes to allow selective interaction of Ncd with spindle microtubules (Fig. 5 D). Without 14-3-3 binding, most Ncd is nonselectively bound and sequestered by numerous nonspindle microtubules in oocytes, resulting in reduced Ncd association with spindle microtubules.

Visual inspection of the Ncd sequence identified a serine (S94) near S96 (Fig. 2 B). This S94 has been shown to be phosphorylated in *Drosophila* (Bodenmiller et al., 2007; Zhai et al., 2008; Hilger et al., 2009) and is potentially phosphorylated by Aurora A/B. We hypothesize that 14-3-3 binding to Ncd is blocked by phosphorylation at S94 by Aurora B, which localizes to the chromosome/kinetochores and the spindle midzone in oocytes (Colombié et al., 2008; Radford et al., 2012b). Thus, this could provide a mechanism to allow Ncd to selectively associate with spindle microtubules around chromosomes.

**Figure 4.**
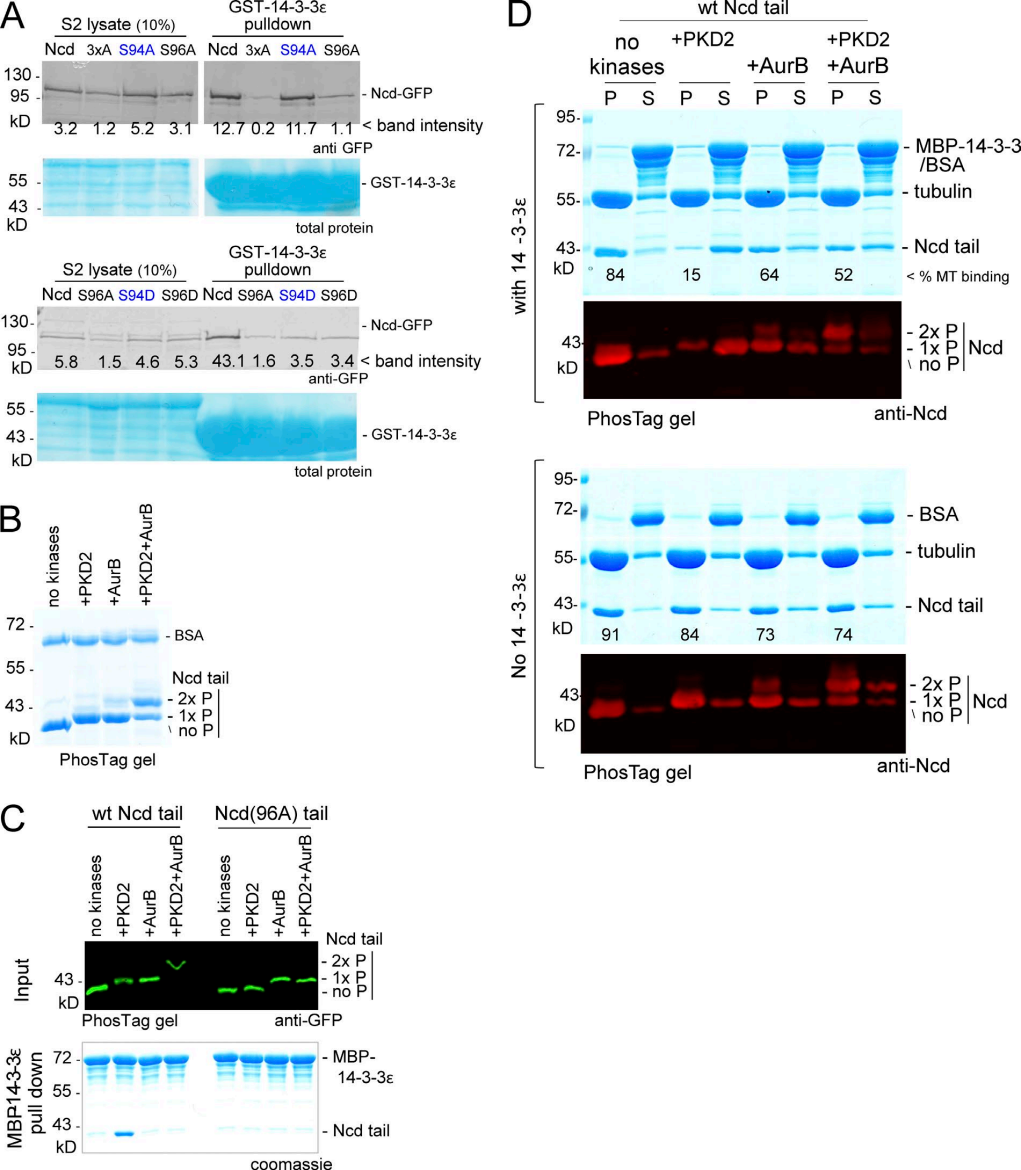
**Aurora B releases Ncd from inhibition by 14-3-3.** (A) Western blot probed by an anti-GFP antibody of pulldown with GST–14-3-3ε from extracts of S2 cells transiently expressing various versions of Ncd-GFP but depleted of the endogenous Ncd. (B) Kinase assay. Coomassie-stained PhosTag gel of the Ncd(58–192) and BSA incubated with kinases. (C) GFP-tagged Ncd tail phosphorylated by kinases (input) and pulled-down fraction with MBP–14-3-3ε. (D) Microtubule (MT) binding assay of differently phosphorylated Ncd tails with or without MBP–14-3-3ε. AurB, Aurora B; P, pellet; S, supernatant.

To test this hypothesis, we first expressed GFP-tagged WT Ncd, nonphosphorylatable Ncd(S94A), and phosphomimetic Ncd(S94D) in S2 cells depleted of endogenous Ncd. Ncd and Ncd(S94A) were efficiently pulled down with 14-3-3ε, whereas the phosphomimetic Ncd(S94D) was not (Fig. 4 A). This suggests that phosphorylation of S94 can prevent 14-3-3ε binding to Ncd.

To test whether Aurora B can phosphorylate Ncd in vitro, we incubated the Ncd tail fragment with Aurora B and/or PKD2. Mobility shifts on a phosphate-affinity (PhosTag) polyacrylamide gel demonstrated that Aurora B phosphorylated the Ncd tail fragment in addition to PKD2 phosphorylation (Fig. 4 B).

To determine the effect of Aurora B phosphorylation on Ncd in vitro, the Ncd tails phosphorylated using these different conditions were tested for interaction with 14-3-3ε or microtubules. Ncd interacted with 14-3-3ε when S96 was phosphorylated, but additional phosphorylation at S94 by Aurora B prevented the interaction (Fig. 4 C). Without phosphorylation, the Ncd tail interacted with microtubules, and S96 phosphorylation by PKD2 inhibited this interaction in the presence of 14-3-3ε (Fig. 4 D) as we previously observed in this study (Fig. 3 D). Further Aurora B phosphorylation restored microtubule binding of Ncd phosphorylated at S96. Aurora B phosphorylation alone slightly reduced the microtubule-binding activity of Ncd independently of 14-3-3ε (Figs. 4 D and S3). These results demonstrated that Aurora B phosphorylation can release Ncd phosphorylated at S96 from the inhibitory effect of 14-3-3ε to allow binding to microtubules.

### Phosphorylation of the putative Aurora B site is important for spindle targeting and function of Ncd in oocytes

**Figure 5.**
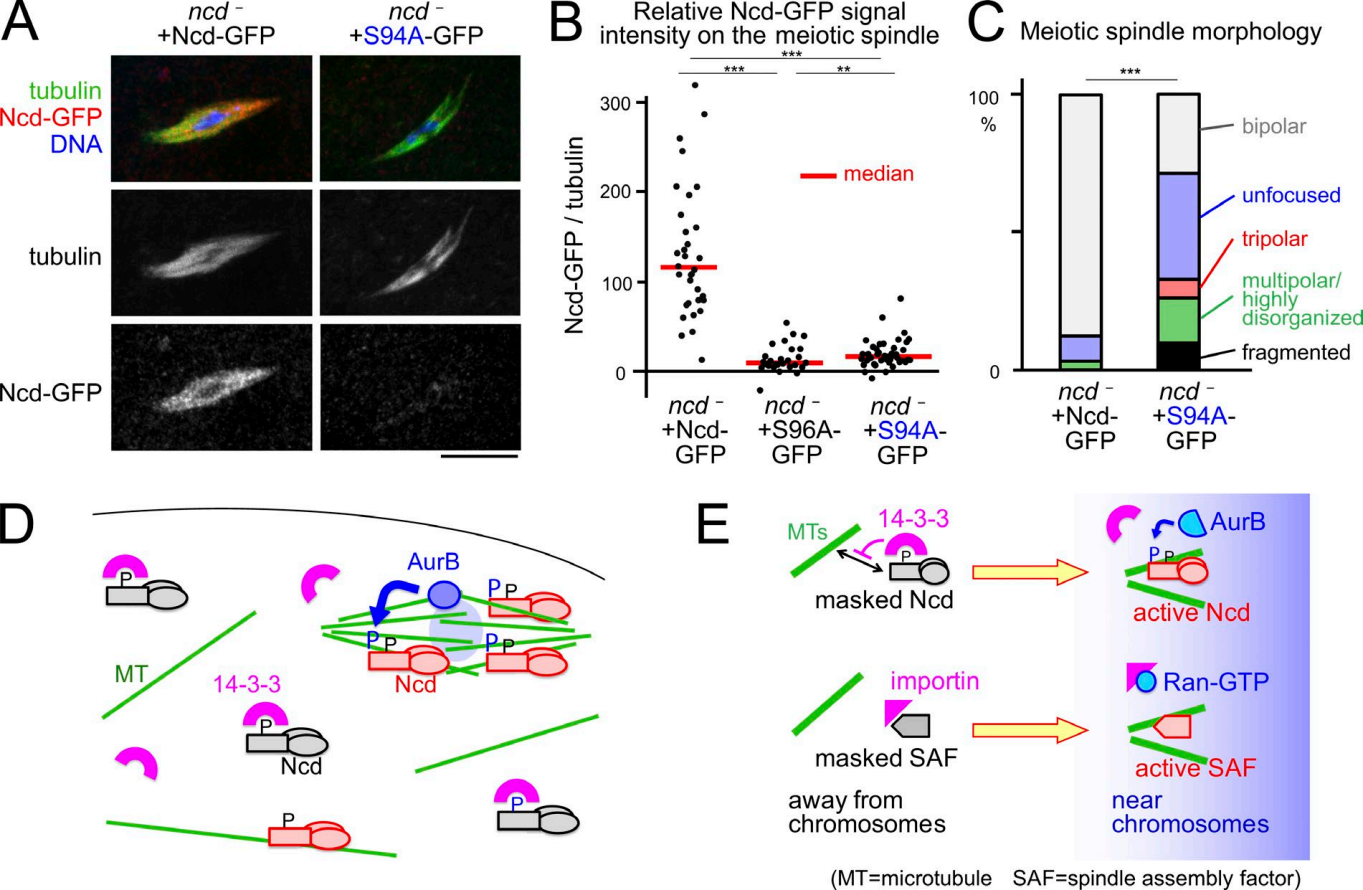
**Aurora B phosphorylation is important for targeting Ncd to the spindle and spindle bipolarity.** (A) Immunostaining of spindles in *ncd^1^* mutant oocytes carrying one Ncd-GFP transgene or one Ncd(S94A)-GFP transgene. Images were captured and modified using an identical setting. Bar, 10 µm. (B) GFP signal intensity relative to α-tubulin signal on the spindle. *n* = 34, 27, and 41. **, P < 0.01; ***, P < 0.001. Note that these were imaged using a different microscope to Fig. 2. (C) Spindle morphologies in oocytes. *n* = 31 and 42. (D and E) Aurora B (AurB) releases Ncd from 14-3-3 inhibition in oocytes analogously to the Ran–importin system, enabling Ncd to specifically bind spindle microtubules.

To test the importance of Aurora B phosphorylation of Ncd at S94 in oocytes, we expressed WT Ncd and nonphosphorylatable Ncd(S94A) in the *ncd* null mutant. The Ncd(S94A)-GFP signal was dramatically reduced on the spindle when compared with the WT version (Fig. 5, A and B) when comparable amounts of protein were expressed (Fig. S1 B). Ncd(S94A)-GFP showed slightly higher residual signal than Ncd(S96A)-GFP, suggesting additional S94-independent regulation. Furthermore, the Ncd(S94A)-GFP transgene did not rescue the spindle abnormalities in the *ncd* null mutant (Fig. 5, A and C). These observations demonstrate that phosphorylation of Ncd at S94 is essential for its efficient spindle association and function in organizing the meiotic spindle.

### 14-3-3 reads the Aurora B spatial cue to selectively target Ncd to the meiotic spindle

Our study has identified a novel mechanism that targets a protein to the meiotic spindle in a large volume of oocytes. 14-3-3 cooperates with Aurora B to target the kinesin-14 Ncd to the meiotic spindle by suppressing binding to nonspindle microtubules (Fig. 5 D).

As oocytes lack centrosomes and are exceptionally large, it is crucial to locally activate the factors important for bipolar spindle formation in response to spatial cues provided by chromosomes. Mainly using *Xenopus laevis* egg extract, the role of the Ran–importin pathway is well described in this context (Carazo-Salas et al., 1999; Kalab et al., 1999; Ohba et al., 1999; Wilde and Zheng, 1999; Gruss et al., 2001; Nachury et al., 2001; Wiese et al., 2001). Importin binds and inhibits proteins collectively called spindle assembly factors, and chromatin-bound Ran-GEF produces a localized signal, Ran-GTP, which releases spindle assembly factors from the inhibitory effects of importin (Fig. 5 E). However, evidence from living mouse and *Drosophila* oocytes suggested the presence of a pathway alternative to Ran–importin (Dumont et al., 2007; Cesario and McKim, 2011). Other studies indicated that chromatin-bound Aurora B is providing a crucial spatial cue in oocytes (Sampath et al., 2004; Colombié et al., 2008; Radford et al., 2012b). How this Aurora B spatial cue translates into local activation of spindle proteins has remained unclear.

Our study uncovered the Aurora B–14-3-3 pathway that acts analogously to the Ran–importin pathway (Fig. 5 E). Away from chromosomes and in a role similar to that of importin, 14-3-3 binds to and inhibits Ncd. Near chromosomes, instead of Ran-GTP, chromatin-bound Aurora B phosphorylates and releases Ncd from the inhibitory effects of 14-3-3. Our study therefore provides a new conceptual framework of how the chromosomal spatial cue is translated into spindle morphogenesis in oocytes.

Evidence suggests that this role of the Aurora B–14-3-3 pathway may be conserved also in mammalian oocytes and that Ncd may not be the only target. Depletion of the mouse 14-3-3η in oocytes results in spindle disorganization (De and Kline, 2013). In mitotic anaphase/telophase mammalian cells, the kinesin-6 MKlp1 is regulated by a similar mechanism (Douglas et al., 2010), which is potentially used also in oocytes. Therefore, the Aurora B–14-3-3 pathway is likely to provide a general and conserved mechanism to locally activate spindle proteins in a large volume of oocytes.

## Materials and methods

### *Drosophila* genetics

Standard fly techniques were used (Ashburner et al., 2005). Controls were WT (*w^1118^*) or flies expressing shRNA against white gene (GL00094) driven using the same driver as in experimental conditions. The following shRNA lines were used: 14-3-3ε line 1 (GL00366), 14-3-3ε line 2 (HMS01229), and 14-3-3ζ (GL01310). The two 14-3-3ε lines gave comparable results (Fig. S1) and were used interchangeably. GAL4 driver lines used were V2H (P{MatTubulin67C-Gal4}V2H), V37 (P{MatTubulin67C-Gal4}V37), and maternal triple driver (MTD; P{otu-GAL4:VP16.R}1; P{GAL4-nos.NGT}40; P{GAL4::VP16-nos.UTR}MVD1). UASp-GFP–α-tubulin recombined with V37 was used for live imaging of the meiotic spindle. For removal of endogenous Ncd in oocytes, flies homozygous for the *ncd^1^* null allele or carrying the *ncd^1^* allele over a deficiency (*Df(3R)BSC547*) uncovering *ncd* were used. These two conditions gave comparable results (Fig. S1) and were used interchangeably. To generate transgenic flies for *ncd-GFP* variants, phiC31 integrase–mediated transgenesis onto the third chromosome was performed by BestGene Inc. using the VK33 site in the strain BL9750.

### Cytology and image analysis

To obtain oocytes, freshly eclosed females were matured at 25°C for 3–5 d on yeasted food in the presence of males. For fixed analysis, samples were dissected in methanol and stained as previously described (Cullen and Ohkura, 2001). Under these conditions, large oocytes consisted mostly of those at stage 14 that naturally arrest in metaphase I. The following primary antibodies were used for immunostaining: anti–α-tubulin (mouse monoclonal DM1A; 1:250; Sigma-Aldrich), anti-TACC (rabbit polyclonal D-TACC–CTD; 1:1,000; Loh et al., 2012), anti-Subito (rat polyclonal; 1:250; Loh et al., 2012), anti-Msps (rabbit polyclonal; HN264; 1:250; Cullen and Ohkura, 2001), anti-Ncd (rabbit polyclonal, 1:1,000; this study), and anti-GFP (rabbit polyclonal; A11122; 1:500 for Fig. 2 or 1:250 for Fig. 5; Thermo Fisher Scientific). Cy3-, Cy5-, or Alexa Fluor 488–conjugated secondary antibodies were used (1:250–1:1,000; The Jackson Laboratory or Molecular Probes), and DNA was stained using 0.4 µg/ml DAPI (Sigma-Aldrich).

Fixed oocytes were imaged as described previously by Głuszek et al. (2015) using an Axioimager attached to an LSM510 Exciter for Figs. 1, 2, and S1 (except the last two bars of Fig. S1 A) or an LSM 800 (ZEISS) for Fig. 5 and the last two bars of Fig. S1 A. Live imaging was performed on a spinning-disk confocal microscope as described previously by Głuszek et al. (2015). Z sections were taken at 0.8 µm at 1-min intervals, and maximum-intensity projections are displayed.

For Figs. 2 and 5, the total signal intensities of tubulin and Ncd on the spindle were estimated using the following formulas: Two areas (L and S) were drawn on the maximum-intensity projection made from Z series of images. Area L includes the spindle and surrounding region, and Area S includes mainly the spindle. We used the formula (I_S_ − N_S_ * [I_L_ − I_S_]/[N_L_ − N_S_])/([I_L_ − I_S_]/[N_L_ − N_S_]), where I and N are the total pixel intensity and pixel number in the specified area, respectively.

To measure the signal intensities of Ncd and α-tubulin along the spindle for Fig. S1, images were taken using identical settings, and maximum-intensity projections were generated. For each spindle, a line was drawn along the spindle length avoiding the region where microtubules are excluded by the chromosomes using the segmented line tool of ImageJ (National Institutes of Health). The gray values of both channels were taken for pixels along the line. A mean background gray value was calculated for each channel from an equal-sized area adjacent to the spindle, and this was subtracted from each value before the Ncd/tubulin intensity values were calculated. These were used to generate a mean value for each spindle. All spindle values were normalized to the median control value in each experiment. To give an idea of staining distribution, pixel intensity data were grouped into 50 sections running from one pole to the other, and mean values were generated for each group. These 50 positions were taken as normalized spindle positions, the values from which could then be averaged for the whole set of spindles for each treatment. P-values were calculated using χ^2^ tests (in the cases of Figs. 1 A, 2 F, and 5 C, the proportion of bipolar to nonbipolar spindles was used) except for Figs. 2 E, 5 B, and S1 D, where the Wilcoxon ranked sum test was used.

### Molecular techniques

Entry plasmids were generated for use with the gateway cloning system by PCR amplifying the desired fragment and introducing it into the pENTR vector using the pENTR Directional Cloning kit (Invitrogen) following the manufacturer’s instructions. To generate the Ncd entry plasmid (Ncd pENTR), the Ncd-coding region without the stop codon was amplified from cDNA (LD29131). S96A mutation, S94D mutation, S94D mutation, and S79A/S96A/S114A triple mutations (3×A) were introduced by PCR amplifying *ncd* fragments using overlapping mutation-bearing primers and joining the PCR products using Gibson assembly (New England Biolabs, Inc.). Mutated plasmids were sequenced to confirm no undesired mutations.

Inserts in the pENTR vector were transferred to the desired destination vector using the LR recombination reaction following the manufacturer’s instructions (Invitrogen). For expression constructs, appropriate destination vectors from the Drosophila Gateway vector collection made by T. Murphy (Carnegie Institution of Washington, Washington, DC) were used. The φPGW and φPWG destination vectors are modified versions of the pPGW Gateway vector into which *attB* had been inserted for PhiC31 integrase–mediated transgenesis.

Transgenic constructs carrying *ncd* variants with the endogenous ncd promoter were generated as follows. SapI sites and additional sequences were added using PCR to both ends of the genomic region containing the 2.8-kb *ncd* upstream region and the 5′ end of the coding region of *ncd*. The additional sequences were designed to have overlaps with the ends of EcoRI–StuI–digested φPWG inserted with the *ncd* coding region. Therefore, the SapI-digested *ncd* promoter fragment was able to be joined together with the EcoRI–StuI–digested *ncd* coding region in φPWG by Gibson assembly.

For RNAi in S2 cells, templates for double-stranded RNA synthesis were amplified using the following primers, and a second PCR step was used to add the full T7 promoter. *ncd* UTRs (forward, 5′-CGACTCACTATAGGGAGACCGTACTCTCCCGACAAATGG-3′, and reverse, 5′-CGACTCACTATAGGGAGACATCGCCAACTGTGTTGTGCC-3′, and forward, 5′-CGACTCACTATAGGGAGATGCATTCTGAGCCCAGTT-3′, and reverse, 5′-CGACTCACTATAGGGAGATTTAGCTTTGAATTCCAGCAC-3′).

GST-Ncd(58–192) in pGEX-6p-2 was used for bacterial expression. The S96A mutation was introduced into this plasmid as described in the first paragraph of this section. The coding sequences from 14-3-3ε cDNA (MIP08648) were cloned into pENTR and then transferred into the pMTWG, pMAL-c2, and pGEX-4T1 destination vectors.

### Protein expression in bacteria and antibodies

For bacterial expression of proteins, bacteria were cultured overnight at 18°C in the presence of 1 mM IPTG. For expression of GST-Ncd(58–192), 0.5 mM IPTG was used, and pellets were frozen and stored at −80°C. To generate the Ncd antibody (Ncd 067), tail-Ncd-His_6_ and stalk-Ncd-His_6_ were purified as described previously (Giet et al., 2002). Proteins were expressed in *Escherichia coli* BL21(DE3) pLysS for 4 h at 25°C, purified on a nickel column, and then dialyzed against PBS. Both purified tail-Ncd-His_6_ and stalk-Ncd-His_6_ proteins were mixed to immunize a rabbit. A phosphospecific antibody designed to recognize pS96-Ncd was generated in rat by Eurogentec (using the peptide KLRRSRpSACDIN coupled to the carrier on the N terminus) as the antigen.

The following primary antibodies were used: anti-GFP (rabbit; 1:1,000; A11122; Thermo Fisher Scientific), anti-Ncd (rabbit; 1:1,000; this study), and anti–pS96-Ncd (rat; 1:1,000; this study). Secondary antibodies were from LI-COR Biosciences and were visualized on an Odyssey scanner (v3.0.30; LI-COR Biosciences) except for right panel of Fig. 3 B, in which HRP-labeled antibodies (Jackson ImmunoResearch Laboratories, Inc.) were used and detected by ECL Western Blotting Detection reagent (GE Healthcare) and films following the manufacturer’s instructions.

### GST–14-3-3ε pulldown assays from S2 cells or ovary extract

For GST–14-3-3ε pulldown, bacteria expressing GST–14-3-3ε or GST control were sonicated in PBS + 1% Triton X-100 + protease inhibitor (cOmplete Mini Protease Inhibitor Cocktail Tablets; Roche) and centrifuged down at 13,000 rpm for 12 min at 4°C. Supernatant was incubated on spinners at 4°C with washed glutathione-Sepharose beads. Beads were subsequently washed three times in lysis buffer (25 mM Tris, pH 7.6, 50 mM NaCl, 1 mM DTT, protease inhibitor, 0.5% Triton X-100, 15 mM Na_3_VO_4,_ 10 mM *p*-nitrophenyl phosphate [alternatively, 1 mM NaF was used], and 1 µM okadaic acid). The beads preloaded with saturating amounts of GST or GST–14-3-3ε were incubated for 2 h with lysed samples prepared from S2 cells or ovaries on a spinner at 4°C. The beads were then washed three times in lysis buffer, then twice in lysis buffer without detergent, and finally were boiled in protein sample buffer.

To prepare S2 cell extract, S2 cells were cultured as described previously (Dzhindzhev et al., 2005). For S2 cell RNAi depletion of Ncd, a mixture of double-stranded RNAs targeting the 5′ and 3′ UTRs of Ncd were used, and 5 d of treatment resulted in the loss of the majority of Ncd protein. For S2 cell transfection, ∼1.5 million cells in a final volume of 1.8 ml were plated in a well of a six-well plate, and plasmids expressing Ncd-GFP under the metallothionein promoter in pMTWG were transfected using X-TremeGENE HP (Roche) following the manufacturer’s instructions. Copper sulfate was added to a final concentration of 0.7 mM. Approximately 5 × 10^7^ cells were pelleted at 500 *g* for 5 min, resuspended in 500 µl lysis buffer, and left on ice for 30 min before being cleared by centrifugation at 13,000 rpm for 30 min at 4°C. To prepare ovary extract, ovaries were dissected at room temperature in PBS + 0.5% Triton X-100 and then transferred immediately to 1 ml of lysis buffer on ice. Approximately 100 ovaries were ground with a glass dounce homogenizer and incubated on ice for 30 min before being cleared by centrifugation at 13,000 rpm for 30 min at 4°C.

### In vitro kinase assay

An in vitro kinase assay using radiolabeled ATP was performed in 10 mM Hepes, pH 7.6, 50 mM KCl, 5 mM MgCl_2_, 1% Triton X-100, 1 mM DTT, 1 mM PMSF, and protease inhibitor (Fig. 3 A). *E. coli* BL21(DE3) pLysS expressing WT or the S96A mutant version of GST-Ncd(58–192) were sonicated in the kinase buffer and spun down, and then 19 µl of the crude lysate was taken. 1 µl of human PKD2 (14-506; EMD Millipore) diluted in kinase buffer to ∼50 ng/µl or 1 µl of human CaMKIIα (BML-SE470) diluted in kinase buffer to ∼50 ng/µl was added. Approximately 5 µCi of [^32^P]γ-ATP was added for 1 h at room temperature, and the sample was boiled in protein sample buffer. The same approach was used for the nonradiolabeled in vitro phosphorylation except that 200 µM of unlabeled ATP was used (Fig. 3 B). For Western blots, proteins were transferred onto nitrocellulose membranes (Amersham Protran 0.2 NC; GE Healthcare) and generally stained for total protein using a Reversible Protein Stain kit for Nitrocellulose Membranes (Thermo Fisher Scientific).

### Mass spectrometry

Protein samples were run on a gel (NuPAGE Novex 4–12% Bis-Tris gel; Thermo Fisher Scientific) in NuPAGE buffer (MES) and visualized using an Imperial Protein Stain (Thermo Fisher Scientific). Gel lanes were cut excluding the maltose-binding protein (MBP)/GST–14-3-3ε band, cut into smaller pieces, destained with ammonium bicarbonate, and shrunk with acetonitrile before being reduced with dithiothreitol and alkylated with iodoacetamide. Buffer containing trypsin (Thermo Fisher Scientific) was used for overnight digestion. Samples were desalted with C18 stage tips (Rappsilber et al., 2007). Peptides were then analyzed using a Velos LTQ Orbitrap (Thermo Fisher Scientific) coupled online to a Dionex RSLC nano system (Thermo Fisher Scientific). Samples were loaded directly onto a column needle self-packed with ReproSil-Pur C18-AQ material (3 µm; Dr Maisch, GmbH) at a flow rate of 0.7 µl/min using a spray emitter (75 µm ID, 8 µm opening, and 300 mm length; new objective) and air pressure pump (Proxeon). For liquid chromatography, a mobile phase A (MQ-H_2_O and 0.1% formic acid) and mobile phase B (80% acetonitrile and 0.1% formic acid) were used. A 60-min gradient was used for the sample set, with a total run time of 100 min per sample. Raw data files were converted to MGF file formats with Convert (Microsoft). Data analysis performed using MASCOT using the following parameters: MASCOT version: 2.4.1; enzyme: trypsin; maximum missed cleavages: 2; peptide tolerance: ±6 ppm; tandem mass spectrometry tolerance: ±0.6 Da; for fixed modifications: carbamidomethylation on cysteine; for variable modifications: oxidation on methionine. The database used was the combined UniProt *Drosophila melanogaster* database (September, 2014). The mass spectrometry proteomics data have been deposited to the ProteomeXchange Consortium via the PRIDE partner repository (Vizcaíno et al., 2016; http://www.ebi.ac.uk/pride) with the dataset identifier PXD006080.

### Protein purification

GST-tagged bacterial expression constructs for the tail region of Ncd (amino acids 58–192) were made in pGEX-6p-2. MBP-tagged bacterial expression constructs for 14-3-3ε were made in pMAL-C2. His-GFP–tagged bacterial expression constructs for full-length Ncd were made in pET-15b. For bacterial expression of proteins, bacteria were grown to OD_600_ = 0.6 and shifted to 18°C before induction with 0.5 mM IPTG overnight. Bacteria were pelleted, washed in PBS, and snap frozen with liquid nitrogen. Pellets were resuspended in lysis buffer containing 50 mM Na-phosphate buffer, pH 7.6, 250 mM KCl, 1 mM MgCl_2_, 5 mM β-mercaptoethanol, and protease inhibitors (cOmplete Mini Protease Inhibitor Cocktail Tablets). Cells were lysed by sonication and clarified by centrifugation at 13,000 rpm for 30 min at 4°C. Supernatant was incubated on a roller at 4°C with either washed glutathione-Sepharose beads or amylose beads for MBP-tagged proteins or with Talon resin for His-tagged proteins for 2 h. Beads were subsequently washed three times in wash buffer (50 mM Na-phosphate buffer, pH 7.6, 250 mM KCl, 1 mM MgCl_2_, 5 mM β-mercaptoethanol, 50 mM arginine, and 50 mM glutamate). Washed beads were transferred to a column, and elution of the protein of interest was done by sequential addition of elution buffer (50 mM Na-phosphate buffer, pH 7.6, 100 mM KCl, 1 mM MgCl_2_, 5 mM β-mercaptoethanol, 50 mM arginine, 50 mM glutamate, 10 mM reduced glutathione [for GST-tagged proteins], 5 mM maltose [for MBP-tagged proteins], and 200 mM imidazole [for His-tagged proteins]). Peak fractions judged by SDS-PAGE were combined and supplemented with 10% glycerol, snap frozen in small aliquots, and stored at −80°C for further use. His-tagged proteins were dialyzed overnight in 50 mM Na-phosphate buffer, pH 7.6, 100 mM KCl, 1 mM MgCl_2_, 5 mM β-mercaptoethanol, 50 mM arginine, and 50 mM glutamate before addition of 10% glycerol and then were snap frozen in small aliquots and stored at −80°C for further use.

### Optimized phosphorylation conditions for the Ncd tail

In vitro phosphorylation of purified WT Ncd or Ncd(S96A) tail was performed in 20 mM Hepes buffer, pH 7.4, 2 mM MgCl_2_, 1 mM ATP, 40 mM KCl, 1 mM DTT, 0.2 mg/ml BSA, and 1 mM EGTA (Figs. 3 D and 4, B–D; and Figs. S2 and S3). Human PKD2 to generate Ncd pS96 was diluted before use in kinase buffer (20 mM Tris, pH 7.5, 50 mM NaCl, and 0.1 mM EGTA) to a final concentration of 25 ng/µl. Human Aurora B (14-835; EMD Millipore) to generate Ncd pS94 was diluted before use in kinase buffer (20 mM Tris, pH 7.5, 50 mM NaCl, and 0.1 mM EGTA) to a final concentration of 100 ng/µl. Ncd substrate to kinase concentration ratio in the final kinase assay was 1:185 for PKD2 and 1:50 for Aurora B. Phosphorylation was performed for 90 min at 30°C and kept on ice until required.

### Microtubule pelleting assays

Taxol-stabilized microtubules were produced by incubating 60 µl of 5 mg/ml porcine tubulin, 6 µl of BRB80 cushion buffer with 40% glycerol, and 1.8 µl of 100 mM GTP at 37°C for 20 min. To stabilize the microtubules, 1.8 µl of 2 mM paclitaxel (taxol) in 110 µl of BRB80 was added to the microtubule polymerization reaction. This tubulin stock mix (17 µM) was stored at room temperature until further use. Proteins to be tested were diluted in the same elution buffer in which they were purified or, in the case of phosphorylation comparison assays, in phosphorylation buffer to give the final concentrations defined in Fig. S2. These protein mixes were then supplemented with BSA to a final concentration of 0.1 mg/ml, and BRB80 containing taxol and microtubules was added to a final concentration of 3.5 µM. To ensure complete binding of 14-3-3 to Ncd, samples were incubated for 5 min before the addition of microtubules. Samples were incubated for 25 min at room temperature to bind microtubules and then were centrifuged for 13,000 rpm for 15 min. The supernatant was removed and mixed with 25 µl 3×SDS-PAGE sample buffer. The pellet was resuspended in 75 µl of 1×SDS-PAGE sample buffer. Equal amounts of both supernatant and pellet fractions were analyzed by SDS-PAGE and Western blotting. PhosTag gel (Wako Pure Chemical Industries) was done according to the manufacturer’s instructions using 25 µM PhosTag and 50 µM MnCl_2_.

### MBP–14-3-3ε pulldown assay of purified Ncd

To test binding of control and phosphorylated versions of either Ncd or Ncd(S96A) to 14-3-3ε, Ncd or Ncd(S96A) were phosphorylated using PKD2 or Aurora B to generate either pS94 or pS96 or both. A final amount of 3.75 µg of Ncd tail was added with 10 µg of MBP–14-3-3ε and diluted in pulldown buffer to a final volume of 500 µl with 25 mM Tris-Cl, pH 7.6, 150 mM NaCl, 0.5% Triton X-100, 0.3 mM NaVO_4_, and 0.1 mg/ml BSA, and then it was incubated on ice for 30 min. 20 µl of amylose resin prewashed and equilibrated in pulldown buffer was added and incubated for 1 h on roller at 4°C. Resin was washed two times with pulldown buffer, and 30 µl of 1.5×SDS-PAGE sample buffer was added and analyzed by either normal SDS-PAGE or 25 µM PhosTag and 50 µM MnCl_2_ for Western blotting.

### Online supplemental material

Fig. S1 shows the effects of 14-3-3 and Ncd on the meiotic spindle in oocytes. Fig. S2 shows how Ncd microtubule-binding affinity is not affected by changes in the charge of S96. Fig. S3 shows how 14-3-3 inhibits Ncd microtubule binding by binding to phosphorylated S96. Table S1 shows mass spectrometry data from 14-3-3ε pulldown.

## Supplementary Material

Supplemental Materials (PDF)

Table S1 (Excel)
