# Supplementary material for: 14-3-3 regulation of Ncd reveals a new mechanism for targeting proteins to the spindle in oocytes
Source: J Cell Biol. 2017 Oct 2;216(10):3029–39. doi: 10.1083/jcb.201704120 (PMC5626551; doi:10.1083/jcb.201704120)
Supplement: Supplemental Materials (PDF) [file JCB_201704120_sm.pdf]

Beaven et al., https://doi.org/10.1083/jcb.201704120

THE JOURNAL OF CELL BIOLOGY

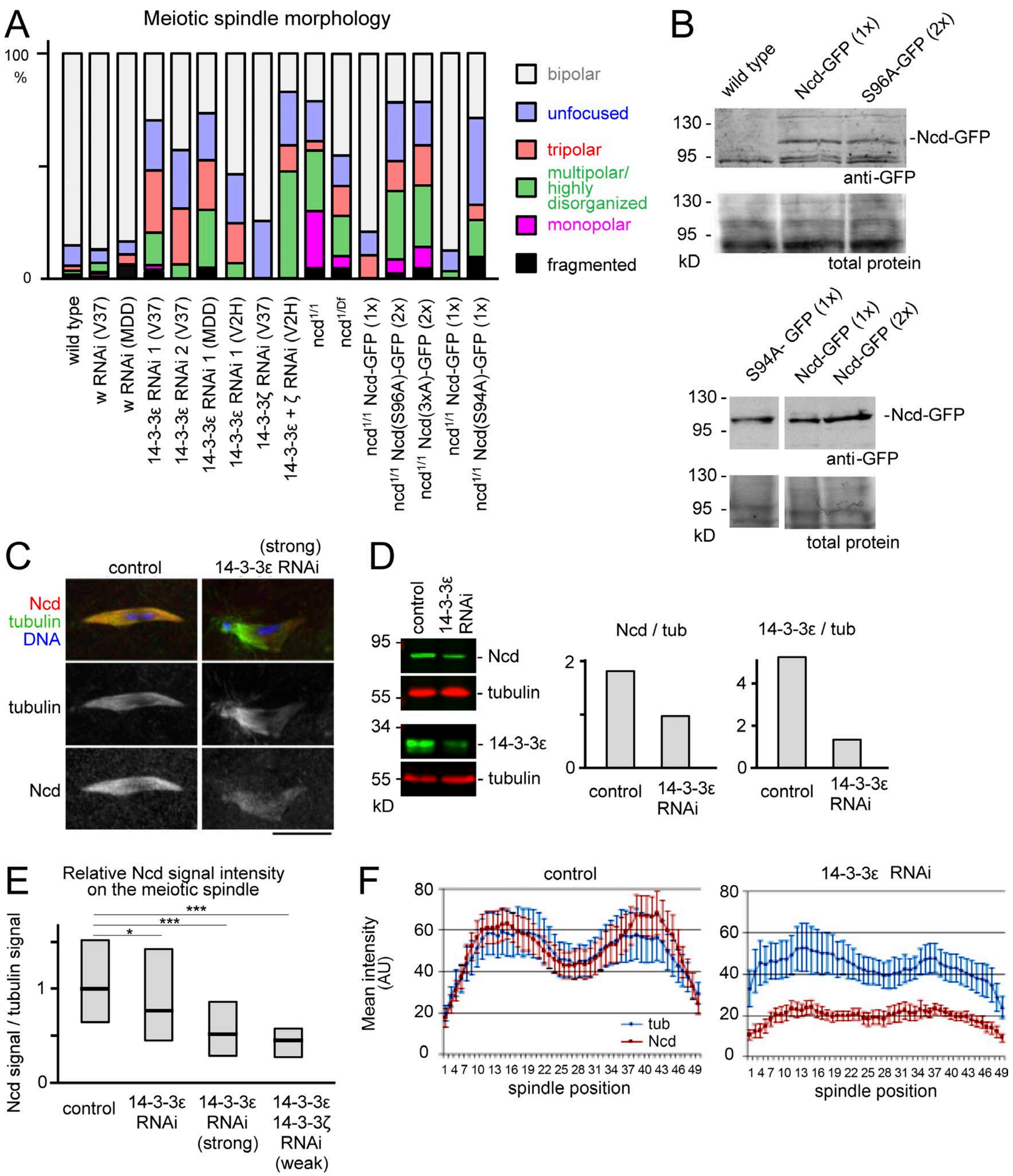


Figure S1. **Effects of 14-3-3 and Ncd on the meiotic spindle in oocytes.** (A) Quantification of spindle morphology in different conditions including the data shown in Figs. 1 A, 2 F, and 5 C. *n* = 59, 19, 135, 39, 44, 33, 16, 17, 32, 23, 22, 13, 10, 50, 22, 31, and 42. (B) Western blot probed with an anti-GFP antibody and total protein staining of ovary samples. One copy of Ncd-GFP, one copy of Ncd(94A)-GFP, and two copies of Ncd(96A)-GFP produced comparable levels of Ncd-GFP variants. (C) Immunostained metaphase I–arrested spindles from control and 14-3-3ε RNAi (using a stronger driver MTD). Images were taken and modified with identical settings. Bar, 10 µm. (D) Western blots of ovary samples and quantification of Ncd or 14-3-3ε signal intensity normalized by tubulin intensity. (E) Ncd signal intensity relative to α-tubulin signal with values normalized to the median control value of each experiment. Strong (MTD) and weak (V2H) drivers were used as well as the routinely used driver (V37). Plots indicate lower quartile, median, and upper quartile. *n* = 159, 37, 117, and 14. *, $P < 0.05$; ***, $P < 0.001$. (F) Distribution of Ncd and α-tubulin signal intensities along spindles (from pole to pole) in oocytes depleted of 14-3-3ε (using the strong driver MTD). Error bars represent SEM. *n* = 17 and 21.

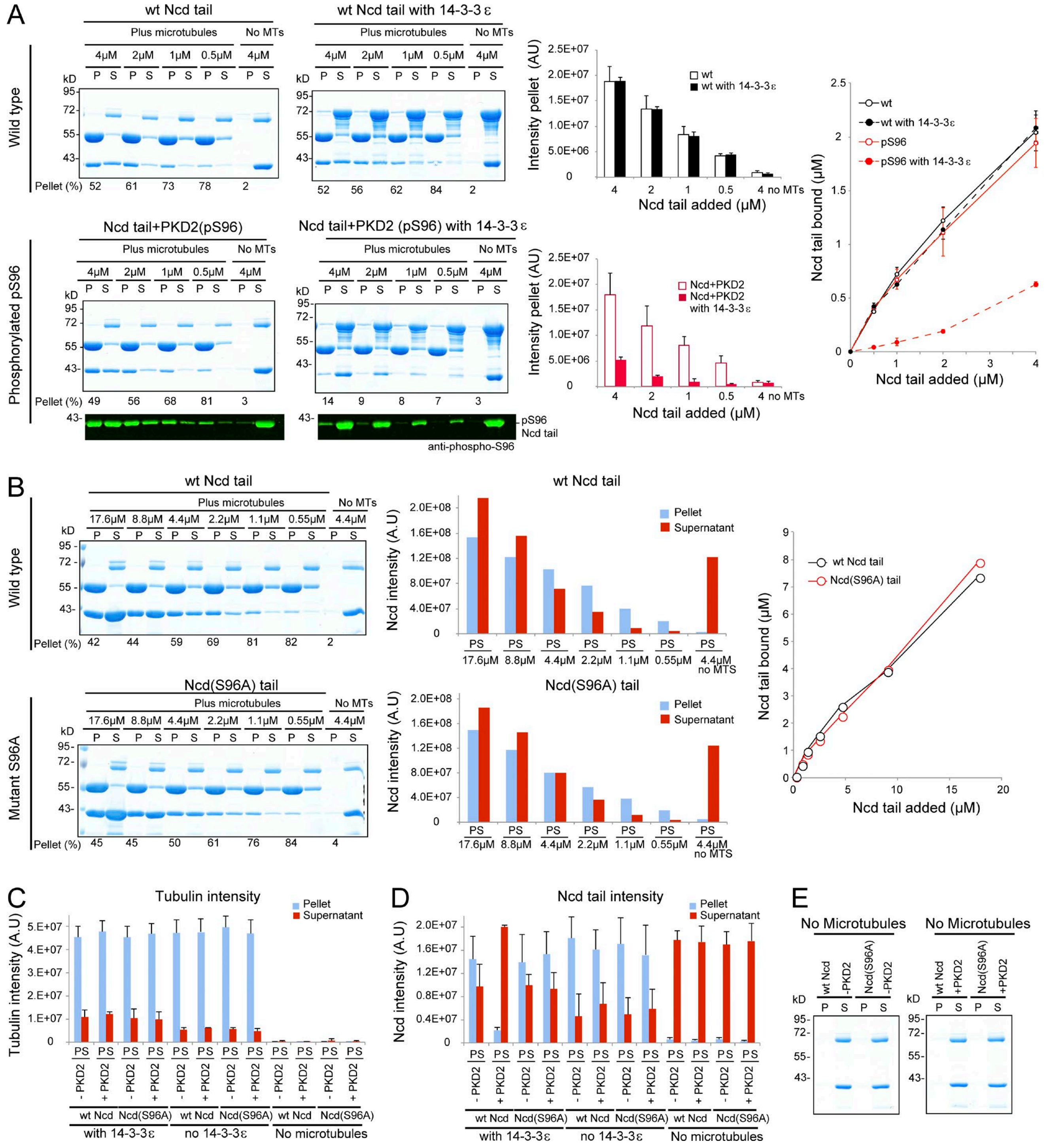

Figure S2. **Ncd microtubule binding affinity is not affected by alanine mutation or phosphorylation at S96.** (A) 14-3-3ε docking to phosphorylated S96 reduced the affinity of the Ncd tail to microtubules (MTs). WT Ncd tail was first incubated with PKD2 to generate phosphorylation on S96 or without PKD2 as control. Then, it was incubated with different concentrations of microtubules and MBP–14-3-3ε (or without MBP–14-3-3ε as control) before spinning down. Pellet (P) and supernatant (S) samples were run on standard SDS gels, which were stained with Coomassie or subjected to Western blotting using the phospho-S96–specific Ncd antibody. The percentage of Ncd tail found in the microtubule pellet is displayed at the bottom of each lane. Intensity of Coomassie-stained Ncd tail in the pellet fractions in either WT Ncd or phosphorylated Ncd (at S96) in the presence (top) or absence (bottom) of 14-3-3ε is displayed in the middle panels. The amount of Ncd tail bound to microtubules as a function of the amount of Ncd tail added is displayed in the right panel. *n* = 3. (B) WT Ncd and Ncd(S96A) tail have the same affinity to microtubules. Variable concentrations of WT Ncd or Ncd(S96A) tail were incubated with microtubules and spun down. The percentage of Ncd tail found in the microtubule pellet is indicated at the bottom of each lane. Quantification of the amount of Ncd tail found in the pellets and supernatant for either WT Ncd or Ncd(S96A) tail is displayed in the middle panels. The right panel displays the amount of Ncd tail bound to microtubules as a function of the amount of Ncd tail added in a representative experiment. *n* = 4. (C and D) Quantification and controls related to Fig. 3 D. Quantification of the Coomassie-stained standard SDS gel shows the amount of tubulin (C) or Ncd tail (D) found in the pellet and supernatant. Error bars represent SD. *n* = 3. (E) WT Ncd and Ncd(S96A) incubated with or without PKD2 in the absence of microtubules are detected in the supernatant in the microtubule-binding assay.

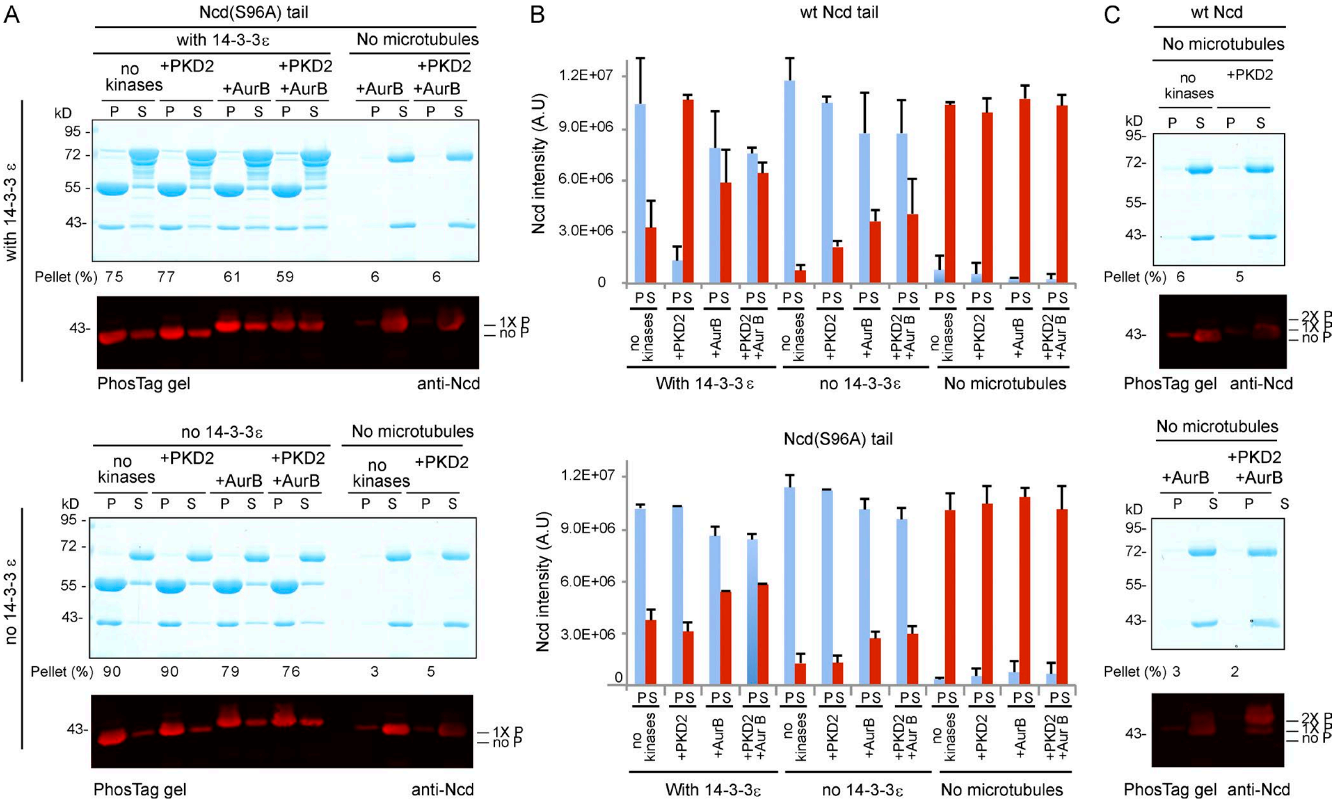


Figure S3. **14-3-3 inhibits Ncd microtubule binding by docking to phosphorylated S96.** (A) Ncd(S96A) binding to microtubules was not disrupted by incubation with PKD2 or Aurora B (AurB) or by the presence of 14-3-3ε. Ncd(S96A) was incubated first with no kinases, PKD2 alone, Aurora B alone, or PKD2 and Aurora B, and then it was incubated with MBP–14-3-3ε before the addition of microtubules before spinning down. Coomassie-stained standard SDS gel of pellet (P) and supernatant (S) samples and PhosTag gel followed by Western blots using the Ncd antibody showing the absence of motility shift when treated with PKD2. The percentages of Ncd tail found in the microtubule pellet are indicated at the bottom of each lane. (B) Quantification of the amount of Ncd tail found in the pellets and supernatant for either WT Ncd (top; related to Fig. 4 D) or Ncd(S96A) tail (bottom; related to A). Error bars represent SD. *n* = 3. (C) Binding control related to Fig. 4 D, WT Ncd incubated with no kinases, PKD2 alone, Aurora B alone, or both was found in the supernatant when microtubules were omitted from the microtubule-binding assay.

**Table S1 is a separate Excel file showing mass spectrometry data from 14-3-3ε pulldowns.**
